# The Management of Congenital Cytomegalovirus Infection in an Era of Universal Newborn CMV Screening

**DOI:** 10.1002/rmv.70132

**Published:** 2026-03-30

**Authors:** Emily R. Harrison, W. Charles Huskins, Mark R. Schleiss

**Affiliations:** ^1^ Division of Infectious Diseases Children's Minnesota St. Paul Minnesota USA; ^2^ Division of Pediatric Infectious Diseases Mayo Clinic College of Medicine and Science Rochester Minnesota USA; ^3^ Division of Pediatric Infectious Diseases University of Minnesota Medical School Minneapolis Minnesota USA

**Keywords:** antiviral, CMV, human cytomegalovirus, newborn screening, sensorineural hearing loss

## Abstract

The most common infectious disease responsible for paediatric developmental disability is congenital infection with human cytomegalovirus (cCMV). Many serious sequelae are caused by cCMV, including microcephaly, intracranial calcifications, neuronal migration defects, seizure disorders, developmental delay, and sensorineural hearing loss (SNHL). Although long‐term neurodevelopmental impairment is clearly more common in newborns with clinically apparent disease at birth, more knowledge is needed about management in the era of universal cCMV screening, since screening identifies infants that, in the past, would not have been discovered during routine newborn care—infants that we reference in this review as having clinically inapparent cCMV (CICMV) infections. For newborns identified with CICMV infections by universal cCMV screening, longitudinal audiologic assessment is needed, but other necessary laboratory and neuroimaging studies, as well as indications for antiviral therapy, are uncertain. This review summarises current concepts about the approach to cCMV infections identified by universal screening, with emphasis on the CICMV infant. We identify areas for future research that should inform and direct future evaluation and management of these infants.

## 1 Introduction

### 1.1 Epidemiology and Transmission

Congenital cytomegalovirus (cCMV) infection is the leading infectious disease‐related cause of developmental disability in children in the United States (U.S.) and Europe [1], and probably globally. An overall global prevalence of 0.7% has been reported in a meta‐analysis, ranging from 0.48% in high‐income countries to 1.42% in low‐and‐middle income countries [2]. In general, there is a positive correlation between the seroprevalence of CMV in the maternal population and the prevalence of cCMV [3, 4, 5]. Primary infections during pregnancy are associated with a 30%–40% risk of vertical transmission [6], and, depending upon the timing of acquisition of infection by the foetus [7], approximately 25% of all infants with cCMV will have long‐term sequelae [8]. Symptomatic cCMV infection can occur both in the setting of primary and non‐primary maternal infections during pregnancy [9]. Kalogeropoulou et al. noted that ‘neurological sequelae are similar following primary or non‐primary maternal infection’ [10], and recent reports from a large universal screening programme in Israel [11] reinforces this data, with at least as many symptomatic and severely symptomatic infections in the non‐primary as in the primary infection group. Overall, most cCMV transmissions that occur in the context of a primary maternal infection occur in the third trimester of pregnancy, and such transmissions are associated with a more favourable prognosis [12]. In the US, health disparities disproportionately impact the likelihood of CMV‐seropositivity, and hence the risk of cCMV transmission, with the highest prevalence rates of infection being noted in black and multiracial infants [13, 14, 15, 16, 17]. Such disparities also contribute to perceptions and acceptance of universal cCMV screening [13, 18], and as universal screening moves forward it will be important to ensure equity in implementation and subsequent follow‐up.

### 1.2 Clinical Presentation and Disease Definitions: Relevance to Universal Newborn Screening

Symptomatic disease due to cCMV has a variety of manifestations. The most severe presentation is that of multi‐organ system disease, also known as cytomegalic inclusion disease (CID), which may present as intrauterine growth retardation; elevation of hepatic transaminases; cholestatic jaundice; dysfunction of the reticuloendothelial system, causing cytopenias, lymphadenopathy, and splenomegaly; dermatologic findings, including petechiae and purpura; and endocrine system abnormalities [19]. Maternal history is an important predictor of symptomatic disease, with a history of seroconversion during pregnancy [20] and/or a history of foetal ultrasonographic abnormalities [21], particularly those noted at or before 20 weeks estimated gestational age (EGA) [22, 23], standing out as important risk factors. Occupational exposure to young children, such as work as a day‐care centre attendant, is a risk factor [24]. However, the current evidence does not suggest that healthcare workers are at an increased risk to acquire CMV infection compared to the general public [25]. A variety of maternal, antenatal, and post‐natal features can be considered in evaluating the risk, and making the diagnosis, of cCMV [26]. These include assessment of maternal serological profile [27], foetal ultrasonographic findings [23], and placental factors [28] that are associated with cCMV. In our clinical practices, we believe it is of value to consider these factors in the evaluation of an infant referred for a positive newborn screening test for cCMV. These are outlined in Table 1. The purpose of this table is to help the clinician to determine the context for a confirmed case of cCMV identified by universal screening.

**TABLE 1:** Maternal, antenatal, and neonatal features associated with congenital CMV infection and disease (after Salome et al. [26]).

Maternal	
History	History of symptomatic maternal infection during pregnancy, including mononucleosis‐like syndrome Occupational exposure in non‐medical setting with extensive exposure to young children (e.g., group day‐care attendants, other childcare setting) [25] Household exposure to young children attending group day‐care centres [73]
Laboratory findings	Documented seroconversion during pregnancy Positive IgM IgG antibody and IgG antibody response with low IgG antibody avidity index [27] Maternal blood PCR positivity [142]
Antenatal	
Ultrasonography	Placental thickening [143] Foetal CNS abnormalities including ventriculomegaly, intracranial calcifications, microcephaly Foetal non‐CNS abnormalities including echogenic bowel, intrauterine growth restriction, hepatomegaly, hepatic calcifications, foetal hydrops [26, 144]
Placental histopathology [28]	Viral cytopathic effect (CMV viral inclusions; immunocytochemistry; PCR) Endothelial damage (RBC extravasation; hemosiderin deposition) Vasculopathy (villous stromal haemorrhage; vascular proliferation and fibrosis) Chronic villitis
Laboratory findings	Amniotic fluid CMV PCR
Postnatal/infant	
Clinical evaluation [19, 26]	Microcephaly (head circumference < −2 SD for gestational age) Petechiae, purpura, blueberry muffin rash Jaundice Hepatomegaly Splenomegaly Abnormal neurologic exam (hypotonia, seizures, abnormal reflexes) Sensorineural hearing loss Ophthalmoscopic findings (chorioretinitis, retinal haemorrhage, optic atrophy, strabismus, cataracts)
Laboratory findings	Anaemia Thrombocytopaenia Leucopenia Isolated neutropenia DNAemia Elevated transaminases (ALT/AST; > 80 IU/mL or > 2 times the upper limit of normal) Conjugated hyperbilirubinemia Abnormal cerebrospinal fluid indices, positive CMV DNA
Neuroimaging findings [19, 26, 33]	
Definitive	Calcifications (often periventricular) Ventricular dilatation Periventricular echogenicity Cortical atrophy Migration disorders Cortical or cerebellar malformations
Indeterminate^a^	Periventricular cysts Subependymal pseudocysts Germinolytic cysts White matter abnormalities Lenticulostriate vasculopathy

^a^ See also Table 3.

**FIGURE 1:**
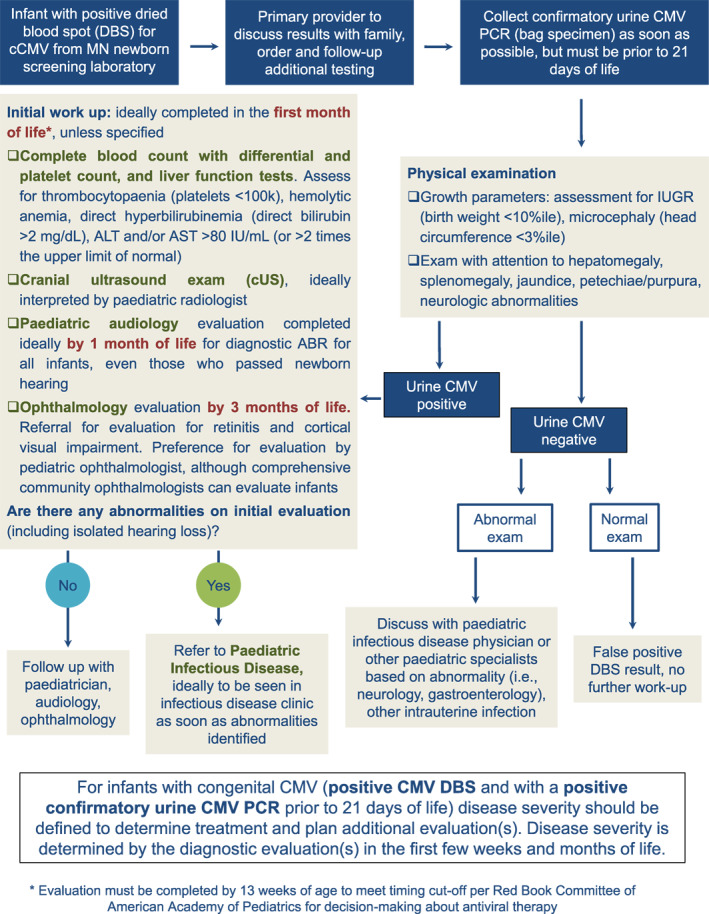
Diagnostic algorithm for infants with positive newborn screening test for cCMV. Outline demonstrates preferred approach of the authors in response to a positive newborn DBS screen for cCMV. As for other screening tests, efforts are made to maximise partnership with primary care physicians, who make the initial communication with the family and coordinate the diagnostic evaluation. We attempt to obtain full diagnostic evaluation in first few weeks of life, with a cut‐off for antiviral decision‐making of 13 weeks of age, per the current Red Book (American Academy of Paediatrics, Committee on Infectious Diseases) criteria (see also Figure 2) [82].

For the congenitally infected infant, a key issue is defining whether cCMV disease is present: that is, is the infection clinically apparent at birth? In this review, we utilise the terms ‘clinically inapparent cCMV (CICMV)’ and ‘clinically apparent cCMV (CACMV)’ in referencing the correlation between the clinical examination of a newborn and the finding of a positive newborn screening test (with confirmation of congenital infection). We use the terms ‘symptomatic’ and ‘asymptomatic’ to refer to clinical conclusions drawn following a complete laboratory, neuroimaging, ophthalmologic and audiologic evaluation, as defined by Rawlinson et al. [19]. A newborn that lacks findings on a standard clinical examination (e.g., microcephaly, seizures, poor tone, hepatomegaly, jaundice, petechiae, etc.) would be considered to have CICMV infection. These infants make up the majority of those detected by newborn screening. However—and importantly—not all CICMV infections are ‘asymptomatic’ ‐ indeed, following an evaluation of such infants (once cCMV infection is confirmed), some are found to have mildly or moderately‐to‐severely symptomatic infection. The findings that are useful in designating disease classification are discussed in greater detail later in this review (in Section 2.2) and outlined in Figure 1. Briefly, the findings in CACMV include signs such as hepatomegaly, splenomegaly, petechiae, retinal disease, and microcephaly. These finding trigger a diagnostic evaluation that is discussed below in Section 2.2 and 2.3; the key difference is that a CACMV infection identified by universal screening is typically an infant who will be identified as having symptomatic disease and would be more likely to have been identified by routine care — even in the absence of a universal screening programme — than the CICMV infant.

### 1.3 Sequelae of cCMV: A Major Driving Force Behind Universal cCMV Screening

The highest rates of long‐term sequelae (ranging from ∼40%–70%) are observed in infants with clinical evidence of infection at birth [29, 30, 31]. Associated clinical outcomes include cerebral palsy, sensorineural hearing loss (SNHL), developmental delay, learning disabilities, seizure disorders, and visual disturbances [32, 33, 34]. Among asymptomatic infants, 1 out of 10 will have SNHL, either present at birth or delayed in onset; in symptomatic infants, the risk of SNHL increases to 1 in 3 children [35]. Since 90% of cCMV infections are asymptomatic/clinically inapparent [36, 37, 38, 39], the true scope of outcomes is likely underappreciated, since clinicians don't typically have a reason to perform cCMV testing on an asymptomatic newborn.

This situation is rapidly changing. In recent years there has been considerable discussion about the question of whether systematic cCMV screening programmes should be implemented into routine newborn care [40, 41, 42], and a number of pilot studies have been performed [43, 44, 45, 46, 47]. With the advent of universal screening for cCMV, clinicians are now poised to identify asymptomatic cCMV infections with increasing frequency. Dollard et al. estimated that among those infants with cCMV that were asymptomatic at birth, 13.5% would have sequelae, whereas for symptomatic infants, 50%–70% would have sequelae: overall, the predicted rate was 17%–20% [29]. Thus, it is important prognostically to draw distinctions between symptomatic and asymptomatic infants. Unanswered questions in making these distinctions include what laboratory and neuroimaging diagnostic evaluations are required; what subspeciality consultations are warranted; what are the appropriate follow‐up studies that are needed; what factors should drive the use of antiviral therapy; and how should parents be counselled about long‐term outcomes?

Accordingly, the goal of this manuscript is to draw from our personal experiences as practitioners providing care for newborns with cCMV to share observations of the challenges presented by universal cCMV screening, whilst providing a review of relevant studies germane to this topic. Drawing from our collective experience in the clinical management of cCMV, particularly in the commonly encountered category of asymptomatic infants that are identified by universal newborn cCMV screening in the state of Minnesota in the US, we offer our perspective on these questions in this review. We note that there is considerable regional variation in practice in the US with respect to the issue of cCMV screening [48]. Additionally, many states in the US have instituted ‘targeted’ cCMV screening (typically driven by a ‘refer’ on the newborn hearing screen [NHS]), and ‘enhanced targeted’ cCMV screening (recommended in the setting of other signs and symptoms of cCMV at birth, noted either in isolation from, or in addition to, an abnormal NHS result), as outlined in a recent review [49], but we limit this current review to our observations and experience with cCMV identified in the course of a universal newborn screening programme.

## 2 Universal Screening: Has It Changed How We Evaluate cCMV?

### 2.1 Historical Perspective and Current Implementation of Universal cCMV Screening

Although recent years have seen intense interest in systematic implementation of newborn screening for cCMV, the idea of making cCMV a screenable disorder is not new. One of the first cCMV screening studies was described by Starr and Gold in 1968 [50]. In this study, pooled newborn urine samples for 562 newborns were assayed by viral culture (the only viral detection technique available at that time) and 8 infants (1.4%) were found to have cCMV; only one infant had symptomatic cCMV. Presciently, the authors commented that ‘…infection seems not to cause serious signs of illness in the majority of newborn infants’ but that ‘…careful neurologic follow‐up will be required to rule out such late sequelae’. Perhaps influenced by the very low rates of cCMV in this early study, data from subsequent cCMV screening studies were interpreted with scepticism with respect to their value. For example, a study from Denmark in 1979 of 3060 neonates, using urine culture, showed an overall incidence of 0.4% with no identified evidence of sequelae in infected infants [51]. Given the ‘very low’ incidence and lack of sequelae, the authors concluded that *‘…CMV infection in our population by no means has a significance to deserve screening’*.

Interest in universal cCMV screening intensified with a multi‐centre study funded by the National Institutes of Health (NIH) in the early 2000s, the CMV and Hearing Multicenter Screening (CHIMES) study [45, 47]. The CHIMES study compared the diagnostic sensitivity of newborn dried blood spot (DBS) PCR with saliva PCR, and the study identified an overall rate of cCMV in the study population of 0.45%–0.55%. Importantly, this study also noted that 43% of all newborns with the eventual diagnosis of cCMV‐related SNHL were not identified via the NHS, hence providing a compelling rationale for universal cCMV screening, since not all cases of SNHL associated with congenital viral infection would be expected to be found by NHS alone [52]. Subsequent to the CHIMES study, the province of Ontario, Canada, recently commenced universal cCMV screening. A recent report spanning July 29, 2019, to July 31, 2023, noted that of 565,987 infants born during the screening period described in this report, 551,034 (97%) had a newborn DBS CMV PCR screening test. Of these infants, 689 (0.13%) screened positive and 601 (87%) had confirmation of cCMV infection, followed by a diagnostic assessment. Among 96 infants with completed assessments, 16% had symptomatic infection, and SNHL was confirmed in 35.4%. The authors advocated for early cCMV identification, both for its potential to improve infant outcomes and in order to reduce unnecessary expense in diagnostic evaluation of older infants presenting with unexplained SNHL [53]. Since this report, universal cCMV screening has been commenced in Saskatchewan, with plans to extend to this to the provinces Alberta and Manitoba [54]. These data are considered in more detail in Section 2.3 of this review (see below).

More recently, universal cCMV screening has been implemented in the US states of Minnesota and Connecticut, commencing in 2023 and 2025, respectively [42, 49, 55, 56, 57]. As in Canada, all universal cCMV screening implemented in the public health arena to date is based on DBS PCR‐based detection of CMV DNA. Although sensitivity may be imperfect, a recent study demonstrated that a positive DBS PCR has an approximate 90% sensitivity in identifying clinically actionable cases of cCMV [57], and since existing infrastructure in state and provincial health departments is ideally suited for DBS CMV detection, the selection of this modality for universal screening is likely to continue into the foreseeable future. Universal cCMV screening in New York was evaluated (funded by the NIH) as a part of a 1‐year pilot programme from 2023–2024, and the data have been recently published [58]. A summary of progress in universal cCMV screening is provided in Table 2, with the key findings and limitations of this newborn screening approach indicated.

**TABLE 2:** Summary of recent findings from state (US) or provincial (Canada) universal cCMV screening programmes [53, 55, 58].

	Ontario (July 2019–July 2023)	Minnesota (Feb 2023–Feb 2024)	New York (Sept 2023–Oct 2024)
Infants screened	565,987 infants	60,115 infants	208,322^b^ infants screened; results reported for 208,099
Screen positivity rate	0.13% (1:800)	0.3% (1:330)	0.3% (1:330) for all CMV (congenital and postnatal CMV or unknown status); 0.13% (1:754) cCMV among newborns screened that had CMV DNA detected in DBS; accounting for false‐positive screens and false‐negative results, final incidence of cCMV was 1:738 of screened population (0.1%)
% Confirmation of cCMV	95.2% (656 infants)	98% (170 infants)	52.2% (276/529)^a^
Complete assessments^c^	87.2% (601 infants)	75% (132/176 infants)	71% (197/279) infants including screen‐negative cases of cCMV completed evaluation; 26 parents declined follow‐up; 36 lost to follow‐up; 17 false‐positive DBS
Symptomatic classification or cCMV disease	16% (96 infants) with ‘symptomatic’ disease classification, described as below: Abnormal physical exam 46/96 infants (48%) Normal physical examination but finding(s) on diagnostic evaluation 50/96 infants (52%) Abnormal cUS 68/96 (71%) SNHL 34/96 (35%)	12% (21 infants) classified as having ‘cCMV disease’ in MDH analysis:^e^ Abnormal cUS in 5/21 (24%) Nonspecific cUS findings in 8/21 (38%) SNHL in 11/21 (52%) Nonspecific cUS findings not included as symptomatic disease	24.6% (68 infants of 276 with confirmed cCMV) had cCMV disease, 3.9% (11 infants) had isolated SNHL. Of 46 infants with neuroimaging findings, 26 of these had no other findings suggestive of cCMV disease.
Treatment with valganciclovir	65.6% (63 of 96) symptomatic infants were treated, with information about therapy decisions in each sub‐group of patients including: CNS disease:^d^ 10/63 2 or more organ systems: 49/63 Isolated SNHL: 2/63 Mild/nonspecific findings: 1/63 Nonspecific neuroimaging findings: 1/63	71.4% (15 infants out of 21 with confirmed disease) were treated with valganciclovir	70.6% (48 out of the 68 infants with symptomatic infection) were treated with valganciclovir

Abbreviation: NA = not available.

^a^ 17/529 were false‐positive DBS screens; positive DBS screens in 236 infants were associated with post‐natal infection, unknown CMV status or infants that were lost to follow‐up.

^b^ Of the 206,725 newborns who were screened, a total of 245 (0.1%) infants were ‘opt out’ of the study, per parental request.

^c^ Complete assessments included complete blood count with differential leucocyte count; platelet count; hepatic function tests; audiologic and ophthalmological evaluation; and neuroimaging.

^d^ Ontario definition of CNS disease: Seizures, microcephaly, imaging abnormalities associated with CMV CNS disease including calcifications, white matter changes, ventriculomegaly, lenticulostriate vasculopathy, cysts.

^e^ MDH disease definition: clinical criteria include hepatomegaly, splenomegaly, petechial rash or purpura, microcephaly, neuroimaging abnormalities consistent with cCMV, sensorineural hearing loss, seizures, cerebral palsy, chorioretinitis, and vision impairment with laboratory confirmation of congenital infection.

### 2.2 The cCMV Screen‐Positive Child: Diagnostic Challenges and Management Dilemmas

The advent of universal cCMV screening has generated many challenges for clinicians. This is not so much an issue for the CACMV infection in a newborn with signs and symptoms of disease identified in the course of clinical care, because there is always a differential diagnosis, including other congenital infections in addition to cCMV, that needs evaluation in this setting [59, 60]. The real dilemma is in the CICMV infant. Indeed, the approach to cCMV in the outpatient paediatric infectious diseases clinic has evolved. In past years, clinic referrals were typically for children with either proven or suspected CACMV (with referrals triggered by clinical manifestations compatible with symptomatic congenital infection). In settings where universal cCMV screening is now in place, we encounter clinical referrals based on positive newborn DBS screening results, absent any signs or symptoms, in children who have CICMV [34]. In Minnesota, the response to a positive newborn screen for cCMV is to empower the primary care physician to manage the initial evaluation of these infants. However, the lack of familiarity with cCMV among health care professionals is a major challenge [61]. Although most primary care providers express interest in learning more about cCMV management, almost three‐quarters indicated in a recent survey that their medical training left them feeling inadequately prepared to manage cCMV [62]. As universal screening programmes move forward in practice, it remains unclear whether the initial evaluation of screen‐positive infants with confirmed infection should be performed by a primary care physician, a specialist in newborn medicine, or a paediatric infectious diseases specialist.

Universal cCMV screening has similarly generated major challenges for families. Prior to the advent of universal screening, the diagnosis of cCMV usually came after a clinically apparent concern prompted a diagnostic evaluation, leading to a cCMV diagnosis. In the context of universal cCMV screening, a diagnosis of cCMV typically takes families and (often) primary care physicians completely by surprise. Parents may feel ‘blindsided’ and can develop a sense of shame and guilt about the diagnosis [63, 64, 65]. We have noted that some families respond with scepticism and denial and decline additional diagnostic evaluation. At the other end of the spectrum, some families react with great fear and anxiety, even for children who have a negative diagnostic evaluation and a normal clinical exam—reinforcing the concern proposed by Gievers et al. about the potential for cCMV screening engendering a ‘vulnerable child syndrome’ [66]. Most families, however, fall between these two ends of the spectrum, expressing appropriate concern but acknowledging that most children do very well, save the small risk of post‐natal and progressive SNHL. Despite some initial distress upon receiving the diagnosis, studies have shown that the long‐term perceptions about newborn screening in cCMV‐affected families are favourable [67].

Once a positive screen is identified, what's next? Below, we offer our recommendations primarily focused on this rapidly evolving area of clinical practice (see also Figure 1). In our experience, the first consultation with a family should be focused on establishing rapport and acknowledging uncertainties in how to approach the diagnostic evaluation, particularly in infants with CICMV infections. As an initial step, all screen‐positive children with presumptive cCMV should undergo definitive virological assessment before 21 days of age to confirm the diagnosis. Diagnosis in this time frame is important since post‐natal acquisition of infection, most commonly through breast‐feeding [1, 68, 69], may lead to viral shedding in the urine by 3 weeks of age, making viral detection beyond this age an unreliable indicator of congenital infection. In cCMV infection, the virus concentrates in the urine, making this an ideal specimen for testing [70]. We recommend a *urine CMV PCR test* for confirmation of cCMV infection in infants with positive newborn screens. Saliva PCR testing to confirm cCMV in a newborn with a positive DBS screen *is not recommended*, since viral DNA must be assumed to be present in colostrum or breastmilk of a CMV‐seropositive lactating mother, making the interpretation of a positive PCR result in an infant exposed to breast milk difficult [71]. Blood/plasma CMV testing is considered diagnostic confirmation for cCMV but is not recommended routinely. In addition to the challenge of obtaining an adequate volume of blood from an infant for testing, while a positive blood PCR may be able to confirm the diagnosis of cCMV, it likely has a lower sensitivity than urine PCR.

The initial history and physical examination can identify infants with clinically apparent disease, but we recommend a comprehensive evaluation of all screen‐positive infants with virologically confirmed cCMV. This recommendation is informed by a study from 2020 [36, 72]. In this study, laboratory, ophthalmologic, neuroimaging, and audiological results of 34 newborns, from Dallas, Texas (US) or Buenos Aires, Argentina were reviewed if they were found to have CICMV infection at birth. Infants were identified based on screening at birth if they fell into a high‐risk category: maternal HIV seropositivity, seroconversion for CMV during pregnancy, sibling with congenital CMV, or a “refer” status on the newborn hearing screen. In spite of these infections being described as CICMV, 56% (19/34) had at least one abnormality noted in these evaluations. The authors commented that these infants would have been classified, based on definitions published by Rawlinson et al. [19], as: 7 infants with moderate‐to‐severe infection (for whom antiviral treatment could be considered); 10 infants with mild symptomatic infection; 3 infants with asymptomatic infection, but with isolated SNHL; and 14 infants with completely asymptomatic infection. The Rawlinson criteria were similarly used to clinically define cCMV categories in infants with confirmed congenital infection in a surveillance study in Minnesota between 2016–2022 [56]. The key point is that a newborn with no clinical evidence of cCMV disease may nonetheless have demonstrable abnormalities after a comprehensive evaluation. As noted above, we draw a distinction between CICMV infection and a ‘symptomatic infection’ defined by the presence of abnormalities when a comprehensive diagnostic evaluation is undertaken: in this context, CICMV infection may, in fact, be ‘symptomatic’ by the Rawlinson definition [36, 73, 74]. Thus, relying on clinical suspicion alone to define whether disease is present or absent is clearly inadequate for defining the scope of cCMV pathologies; making decisions about therapy; and, perhaps, for counselling families about the future prognosis for newborns identified in universal screening programmes [75, 76].

As shown in Figure 1, the diagnostic evaluation should consist of: hepatic function tests; complete blood count with platelet count and differential leucocyte count; a cranial ultrasound (cUS) examination; an ophthalmologic evaluation; and a detailed audiologic assessment. The laboratory studies are focused on examination for thrombocytopaenia (< 100,000/mL) and anaemia; the liver function tests, to assess for direct hyperbilirubinemia (direct bilirubin > 2 mg/dL), and ALT and/or AST > 80 IU/mL (or greater than 2 times the upper limit of normal). In infants with symptomatic cCMV the bilirubin and transaminases are described to peak in the first 2 weeks of life but then can remain elevated for several weeks. Similarly, in infants with symptomatic cCMV, thrombocytopaenia may reach a nadir by approximately 2 weeks of age and then may normalise by 3–5 weeks of life [37, 38]. The role of checking blood and/or urine CMV viral load as a component of the initial diagnostic evaluation is not well established. Studies have suggested that higher baseline CMV viral loads are associated with symptomatic disease [77, 78] and hearing loss; however, no viral load breakpoint predictive of hearing loss has been established. It is not our routine practice to check quantitative CMV viral load from blood, as parental counselling regarding specific values can be difficult. Since viral load determination does not affect the disease categorisation of infants with cCMV (described below), this parameter does not impact patient management.

A detailed audiologic evaluation is a key component of the diagnostic rubric. The results of the NHS, whether it is scored as a ‘pass’ or a ‘refer’, cannot be used to fully characterise hearing function; rather, a bona fide diagnostic auditory evoked response (ABR) study is required. Children with cCMV often have concomitant vestibular dysfunction (considered in more detail below), but the challenges of performing paediatric vestibular testing make this an impractical consideration for routine testing at the current time. Although controversial, we also recommend ophthalmologic evaluation in all infants with cCMV, including those with clinically inapparent infections, since newborns with CICMV may rarely but importantly demonstrate ophthalmologic findings, and we have identified unexpected (but clinically important) ophthalmologic findings, including retinal scarring, as the sole manifestation of potential cCMV disease in our universal newborn screening experience (https://cmv.usu.edu/schedule/grid‐details.cfm?pg=none&aid=18515&ts=3788&ty=grid&des=reg accessed March 17, 2026).

Evaluation should be performed in a timely manner, since initiation of antiviral therapy (for those infants who are candidates for treatment) is time‐sensitive. This is discussed in more detail below in Section 3.2. In the only 2 randomized controlled trials on treatment for symptomatic cCMV, antiviral therapy was started in the first month of life [79, 80]. There is a more recent non‐randomized trial of infants with CICMV and isolated SNHL that suggests there is benefit if treatment is started beyond the 4^th^ week of life, extending potentially out to 13 weeks of age [81]. Based on these studies, the goal is to identify symptomatic infants by 4 weeks, irrespective of whether their cCMV infections are clinically apparent or inapparent, but the clinical evaluation must be completed at the very latest by 13 weeks of age, so that these infants may potentially benefit from initiation of antiviral treatment to ameliorate the risk of SNHL [81].

### 2.3 The Screen‐Positive Child With Proven cCMV: Disease Categorisation and Neuroimaging Interpretation Complicates Comparisons of Newborn Screening Programmes

We recommend using the Rawlinson et al. criteria [19], which are essentially identical to those outlined in the 2024–2027 Red Book [82], in determination of the cCMV disease classification in infants identified with cCMV infection in the context of universal screening programmes. However, interpretation of neuroimaging findings, particularly cUS findings, has been demonstrated to be a source of confusion and uncertainty. Of particular interest are non‐classical findings that are not clearly indicative of central nervous system (CNS) pathology. Findings more clearly recognized to be associated with symptomatic cCMV (which may or may not be clinically apparent) include intracranial calcifications (often periventricular), periventricular echogenicity, intracranial ventriculomegaly without other explanation, cortical or cerebellar malformations, white matter changes, and neuronal migration abnormalities [8, 19, 32]. However, we have commonly observed several cUS findings that may not constitute clinically significant CMV‐induced lesions; hence, in our view, they are of uncertain significance. These include subependymal cysts; germinal matrix cysts; choroid plexus cysts; grade I germinal matrix haemorrhage; and lenticulostriate vasculopathy (LSV) [33, 55]. Further complicating this issue is the fact that current diagnostic classification schemes have inconsistent criteria regarding the findings that define CNS involvement due to cCMV. The European Congenital CMV Infection Initiative [77], the European Society of Paediatric Infectious Diseases CMV Working Group [83], and the Canadian Paediatric Society [https://cps.ca/en/documents/position/update‐on‐congenital‐cytomegalovirus‐infection‐prenatal‐prevention‐newborn‐diagnosis‐and‐management] include findings such as LSV and subependymal/germinal matrix/choroid plexus cysts as indicative of cCMV with CNS involvement, while the Rawlinson et al. working group [19], the Red Book committee of the American Academy of Paediatrics [82], and the CDC's Council of State and Territorial Epidemiologists (CTSE) have more stringent criteria [https://ndc.services.cdc.gov/case‐definitions/congenital‐cytomegalovirus‐ccmv‐infection‐and‐disease‐2024/] for defining whether neuroimaging findings are cCMV‐related (Table 3). More work is needed before concluding that many of these neuroimaging findings that we characterise as variants of uncertain significance [8, 33], such as LSV and various types of cysts, are truly related to cCMV neuropathology, and in particular before basing antiviral therapeutic decisions on these findings, especially if these occur in isolation from other signs, symptoms neuroimaging and laboratory markers of symptomatic cCMV disease.

**TABLE 3:** Summary of neuroimaging findings considered indicative of cCMV with CNS involvement according to different expert groups (after Kruc et al. [33]).

	Rawlinson et al. (cCMV international working group; [19])	Luck et al. (European society of paediatric infectious diseases working group; [83])	Leruez‐Ville et al. (European congenital CMV infection initiative; [77])	American academy of paediatrics 2024–2027 red book committee [82]	Canadian paediatric society	Council of state and territorial epidemiologists (CSTE) case definition for CMV disease
Neuroimaging findings (as stated)						
Ventriculomegaly	X		X	X	X	X
Ventricular dilatation		X				
Intracerebral calcifications	X	X	X	X	X	X
Periventricular calcifications			X		X	
Periventricular cysts		X	X			
Periventricular echogenicity		X		X		
Periventricular leukomalacia						X
Brain atrophy			X			
Cortical malformations	X		X	X		
Cortical atrophy		X				
Cortical hypoplasia		X				
Cerebellar malformations	X			X		
Cerebellar hypoplasia			X			
Dysgenesis of the corpus callosum			X			
Subependymal pseudocysts		X				
Germinolytic cysts		X	X			
Occipital horn septations			X			
Cerebellar, ependymal, parenchymal cysts					X	
White matter abnormalities		X	X	X		
Migration disorders (polymicrogyria, lissencephaly, porencephaly, Pachygyria, schizencephaly)		X	X	X	X	X
Extensive encephalopathy					X	
Lenticulostriate vasculopathy		U	X		U	

Abbreviations: X = indicative, U = uncertain.

For infants with CICMV or minimally symptomatic findings who have findings of uncertain significance on cUS, the question of when to pursue additional head imaging with an MRI can be a complicated one. Some of the key issues include: (1) Will the findings of an MRI add to decision‐making with respect to the use and duration of antiviral therapy? (2) Can an MRI in a neonate give better prognostic information regarding long‐term outcomes? (3) Does a normal cUS provide adequate reassurance, or do some patients with a normal cUS need follow up with an MRI? (4) If MRI is performed, what is the optimal timing for that exam? Alarcon et al. has developed a cUS and brain MRI based scoring system that attempts to predict outcome in the setting of cCMV; however, this has been studied most extensively in infants with symptomatic cCMV disease [84]. A 2024 retrospective cohort study applying this scoring system included assessment of 45 asymptomatic infants; imaging abnormalities were identified in otherwise asymptomatic infants but overall lower scores were noted than in those with symptomatic disease and only 3 otherwise asymptomatic children with imaging abnormalities progressed to moderate or severe disability [84]. While such a scoring system may be helpful for determining the overall neurodevelopmental prognosis, there is less clear association between score and outcome in asymptomatic infants and there are no studies indicating at which threshold of abnormality an infant may benefit from antiviral treatment, limiting the current clinical utility of this algorithm.

Since our current practice is to begin with cUS, we take a selective approach in formulating our recommendations for brain MRI testing. Not every infant identified by universal cCMV screening requires a brain MRI. MRI is useful if cUS findings are noted, in particular if the findings are not pathognomonic for cCMV pathology; in such cases, MRI can clarify uncertainties [33]. Moreover, when an MRI is recommended, a full, sedated study is not always required. A rapid‐sequence brain MRI (also known as a ‘quick‐brain’ or ‘fast‐brain’ study) is usually adequate [85], and does not require sedation and/or hospitalisation. With respect to those infants for whom a brain MRI is routinely recommended, we propose that infants with abnormal audiological findings are of a particularly high priority for this study on a routine basis. A study of a small cohort of 17 children with cCMV who failed their newborn hearing screen showed that 59% had an abnormal brain MRI [86]. We do not, however, recommend brain MRI for confirmed cCMV in newborn screen‐positive infants solely on the basis of a ‘refer’ status on the NHS; however, if demonstrable SNHL is found, this study is warranted. In addition, we found in a prospective screening study of cCMV in Minnesota that variants of uncertain significance on cUS were more common among infants that had other cCMV‐related manifestations noted on their diagnostic evaluations, particularly among infants that had asymptomatic infection with isolated SNHL; those that qualified as having “mild cCMV disease”; and those with moderately‐to‐severely symptomatic cCMV disease but without typical neuropathologies as defined by Rawlinson et al. In total, 9/13 of these infants had variants of uncertain significance by cUS; 69%). In contrast, for infants that were categorised as asymptomatic cCMV infection (with normal hearing), only 9/58 (16%) had such incidental cUS findings (*p* < 0.0001) [33]. Thus, for infants with normal hearing and a negative laboratory diagnostic evaluation, and who have a normal cUS, we do not feel routine brain MRI is routinely warranted, based on our current level of understanding, but it can be considered if variants of unknown significance are encountered by cUS in the presence of other signs, symptoms and laboratory findings. This is an important area for future investigation [87, 88, 89, 90]. We do suggest that in infants that demonstrate documented SNHL following a diagnostic audiological evaluation (not just a ‘refer’ on newborn hearing screen), that a brain MRI may have particular value [91], and we do typically recommend this. It is important to coordinate this carefully with our paediatric otolaryngology colleagues. If a brain MRI is to be obtained for an infant with SNHL, it is imperative that internal auditory canal sequences also be obtained, as this is a requirement for later cochlear implant surgery. Otherwise, these infants may need to repeat the MRI later in infancy just to obtain that additional imaging. Getting all the imaging data that may be helpful for the long‐term management of SNHL, in a single imaging examination, is certainly in the best interest of the infant.

The range of clinical findings upon diagnostic evaluation of infants identified by universal screening, as well as information regarding the use of antiviral therapy, has been described by public health representatives in Ontario and Minnesota following implementation of their cCMV screening programmes, and is shown in Table 2. In Ontario's newborn screening programme sixty‐three infants were symptomatic and treated with valganciclovir (10 for significant CNS disease only; 49 for manifestations in 2 or more systems; 2 for isolated SNHL; 1 for mild non‐specific findings; and 1 for non‐specific neuroimaging findings). In Minnesota, a pilot study of cCMV from 2016–2022 of ~25,000 newborns, using the Rawlinson et al. criteria, observed that 17.2% of infants had symptomatic cCMV disease at birth (excluding isolated SNHL in otherwise‐asymptomatic infants) [57]. Following formal adoption of universal cCMV screening by the MDH, a report of the first year of screening noted that, of 176 confirmed cases, 21 (12%) met the published CMV case definition of ‘CMV disease’ as defined by the CSTE in the US (https://ndc.services.cdc.gov/case‐definitions/congenital‐cytomegalovirus‐ccmv‐infection‐and‐disease‐2024/) [92]; the Rawlinson criteria were not used. The Minnesota investigators noted that, in many cases, what they characterised as ‘nonspecific neuroimaging abnormalities’ did not result in a determination of ‘symptomatic disease’. In the recent report from New York [58], among the 276 newborns with cCMV, a surprising observation was that 68 (24.6%) of these had symptomatic cCMV disease. In total, 48 out of the 68 infants identified in this study with symptomatic cCMV infection were treated with valganciclovir. In contrast, the Ontario study identified symptomatic cCMV in a lower rate of cCMV infants ‐ 96/601 infants (16%). The New York investigators noted this difference, stating that ‘the reason for the difference is unclear’, but it seems likely that the differences are attributable to differences in interpretation of cranial imaging findings. The New York study methodology used the Rawlinson criteria for initial identification of cCMV disease classification, but noted later in the manuscript that twenty‐four of 46 newborns with any neuroimaging finding (and therefore 24 of the 68 total symptomatic infants) were determined to have symptomatic cCMV disease based only on imaging studies [58]. If the 24 newborns with non‐specific cUS findings were excluded, the rate of symptomatic cCMV cases (excluding asymptomatic cCMV cases with isolated SNHL) would be 44/276, or 16% ‐ in keeping with the Ontario [53] and Minnesota [57] studies. A similarity between the Ontario and New York surveillance programs is the overall lower‐than‐expected cCMV detection rate (0.1%), which was identified in both screening programmes. In contrast, the Minnesota detection rate during the surveillance study was 0.37% [57], and in the first year following introduction of CMV to the routine newborn screening programme, approximately 0.3% [55]. The reasons for the unexpectedly low prevalence in New York and Ontario require further investigation, as does the unresolved question of the relative sensitivity of DBS PCR testing compared to other screening modalities—although reassuringly in the Minnesota pilot study, the clinical sensitivity of DBS PCR (compared to saliva) for identification of cCMV in infants who had symptoms and sequelae from their congenital infections was reported to be in the range of ∼80–90%, depending upon the PCR assay used [57]. This provides reassurance that although the analytic sensitivity of DBS may not yet be optimal, that it does have the ability to discriminate those congenital infections that are associated with potential long‐term sequelae of cCMV.

## 3 cCMV Identified by Universal Screening: Management

### 3.1 A Time‐Sensitive Need to Define the Extent of cCMV Disease

A key management challenge as universal cCMV screening moves forward is the time sensitivity of clinical decision‐making. The situation is further complicated by the fact that cCMV‐associated SNHL is commonly delayed in onset, making these disease definitions age‐dependent for some infants [35, 93]. Even in infants with SNHL identified in the newborn period, timing of initiation of antiviral therapy is critical. A CASG study (ClinicalTrials.gov identifier NCT01649869) of the potential benefit of valganciclovir therapy initiated after 1 month of life (at a median of 18.7 months in this study) failed to demonstrate any benefit of treatment. On the other hand, the CONCERT study (discussed in more detail in following section), in which therapy was initiated at median age of 8 weeks, demonstrated a benefit against hearing deterioration at 18 through 22 months of age in treated children compared to untreated controls [81]. These conflicting studies suggest that the ideal timing of initiation of antiviral treatment is a key question to be resolved as we move forward with universal screening programmes [8, 19, 33, 77, 81, 82, 83, 94, 95, 96, 97]. Our current recommendation is to follow the Rawlinson et al. criteria [19] with respect to diagnostic categorisation and antiviral therapy recommendations, recognising that the advent of universal cCMV screening has complicated our past definitions of cCMV disease. We temper all recommendations with shared decision‐making after consultation with families, as also recommended by the Red Book committee [82], and as we outline below.

### 3.2 Antiviral Therapy

**FIGURE 2:**
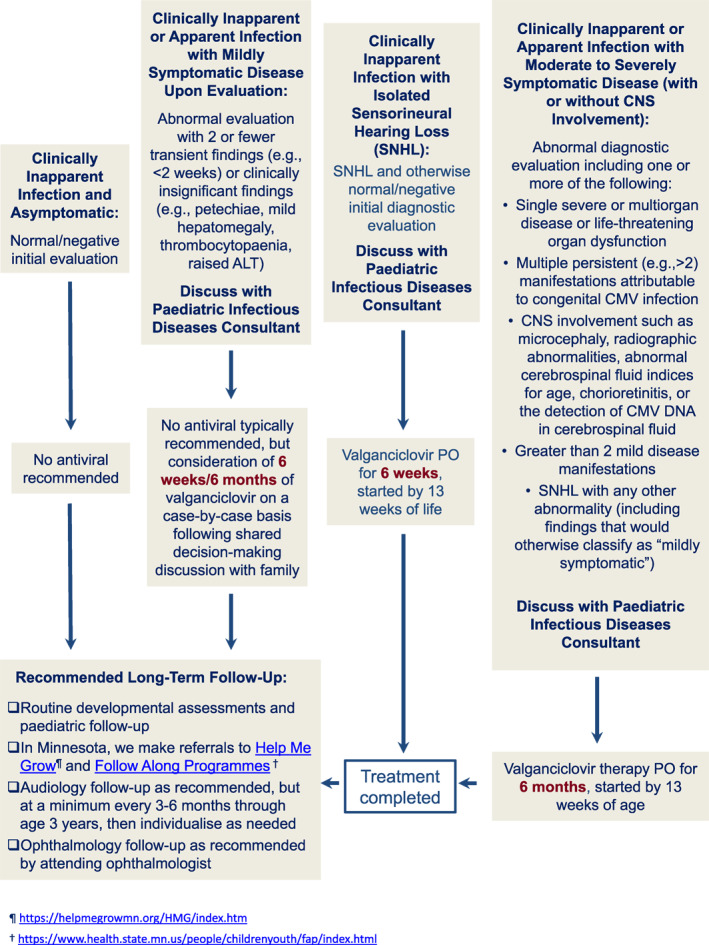
Therapeutic approach for infants with positive newborn screening test for cCMV in Minnesota. Figure demonstrates our preferred approach in managing a confirmed case of cCMV. In collaboration with the infant's primary care physician and audiologist, treatment algorithm parallels recommendation of Red Book [82]. Discussion points with family and details for monitoring are as outlined in the text. Links are provided for interest of the readership for Minnesota's ‘Help Me Grow’ and ‘Follow Along’ programs, which are recommended for all cCMV infected infants, irrespective of disease category and/or treatment details.

As noted in a recent scoping review of the topic by Boscarino et al. [98], although there has been a steady increase in published reports of the use of antiviral therapy in the management of infants with cCMV, there is a paucity of controlled trials. A review by Pata et al. drew similar conclusions [99]. We base our recommendations for antiviral therapy (summarised in Figure 2) on the foundation that was established by two historical multicenter studies conducted by the Collaborative Antiviral Study Group (CASG). The first was a randomized placebo‐controlled trial of 6 weeks of intravenous ganciclovir for infants with cCMV‐associated central nervous system (CNS) disease. The study documented reduced hearing loss at 6 months in ganciclovir‐treated infants [79]. A follow‐up study was a pharmacokinetic/pharmacodynamic study that demonstrated that oral valganciclovir (the prodrug of ganciclovir) at a dose of 16 mg/mg achieved plasma concentrations similar to that of intravenous ganciclovir [100].

These studies served as the basis for a second pivotal trial, a subsequent randomized, controlled trial (RCT) conducted by the CASG, the CASG 112 study. This study compared 6 months versus 6 weeks of oral valganciclovir (16 mg/kg/dose twice daily) started within the first month of life for treatment of infants with moderately‐to‐severely symptomatic cCMV [80]. This was a relatively small study with fewer than 50 subjects in each treatment arm. There was a high burden of disease in these CMV‐infected newborns, with most infants having neuroimaging abnormalities and approximately one‐third with SNHL. Hepatosplenomegaly, hepatitis, and thrombocytopaenia were common, and many infants had more than one abnormality. Both treatment groups demonstrated a significant decrease in whole‐blood CMV DNA viral load during the first 6 weeks of treatment. The 6‐month treatment group maintained this decrease during the entire 6‐month treatment period, whereas the 6‐week treatment group had a significant rebound after 6 weeks that continued over the next several months. The primary outcome was the change in ‘best ear’ hearing from baseline to 6 months of age as determined by an audiologist blinded to treatment assignment. There was no difference between the treatment groups in the primary outcome at 6 months, a finding that was not changed by a prescribed secondary analysis adjusting for baseline CNS involvement. However, examining other prescribed secondary outcomes, the 6‐month treatment group demonstrated better hearing outcomes after adjustment for baseline CNS involvement and modestly better developmental scores at both 12 and 24 months. The timing of initiation of treatment (< 3 weeks vs. 3–4 weeks of life) did not correlate with hearing outcomes. Significant neutropenia was a common adverse effect (approximately 20%) in both groups throughout the 6 months. This study provides the rationale for the Red Book recommendation for 6 months of valganciclovir treatment for newborns with moderate to severe symptomatic cCMV with or without CNS involvement [82], with the additional statement that treatment should be started within the first 13 weeks after birth (see below).

A major subsequent question has been whether there is a benefit of treatment for less severely affected infants, such as those with some degree of SNHL as the only significant finding after a standard evaluation [101]. A definitive answer to this question remains to be identified, but several recent studies have addressed this. A RCT of 6 months of valganciclovir versus placebo on progression of hearing outcomes in neonates with SNHL only (NCT03107871; https://www.clinicaltrials.gov/study/NCT03107871) was stopped due to difficulty with recruitment [102]. Another RCT evaluating 6 weeks of valganciclovir started in infants older than 1 month of age on the change in ‘total ear’ hearing between baseline and 6 months found no effect [103]. However, the mean age at enrollment in this study was 18.7 months, so it is plausible that treatment began later than at an age at which a benefit of treatment is likely. On the other hand, a non‐randomized trial (CONCERT 2.0) of 6 weeks of valganciclovir versus no treatment of infants with SNHL and clinically inapparent cCMV found that treatment reduced hearing loss at 18–22 months of age [81]. While the study examined outcomes in infants in whom treatment was started within 13 weeks of life, the majority of infants started treatment earlier (median age of 8 weeks at the start of treatment, interquartile range 6–10.5 weeks). Based on this result with a broader timeframe for initiation of treatment, the Red Book has now indicated that parents of infants with isolated SNHL may be offered a 6‐week course of valganciclovir treatment started within 13 weeks after birth [82]. The recommendation to start treatment by 13 weeks of life was expanded to children with moderate to severely symptomatic disease as well, despite the paucity of data in this group.

We also note, and agree with, the Red Book recommendation that parents may also be offered valganciclovir in infants with only ‘mildly symptomatic disease’ (no CNS involvement, no evidence of SNHL at birth or delayed in nature). We note that the Red Book committee does not provide any specific guidelines for the duration of antiviral therapy in this setting [82]. It should be noted that the CONCERT 2.0 study on which the 6‐week recommendation appears to be based did not compare 6 weeks to 6 months, only comparing a 6‐week course to no treatment [81]. Some experts suggest administering 6 weeks of antiviral therapy for mild disease (no CNS involvement) or isolated SNHL, but allowing for a range from 6 weeks to 6 months, given the fact that the only data comparing duration of therapy was performed (in the CASG 112 study [80]) on newborns with relatively severe disease and appeared to favour longer treatment. We also acknowledge the recommendation from Rubinacci et al. for infants with isolated thrombocytopaenia or hepatitis in which, absent any other signs, symptoms or aberrant laboratory or neuroimaging abnormalities, that a 6‐week duration of oral valganciclovir (Figure 2) would be an appropriate course of therapy [104], although the evidence base for this recommendation is less clear.

Families of infants with asymptomatic cCMV commonly ask about antiviral therapy, with the hope that treatment may mitigate even the very minimal risk of sequelae in this setting. A Phase 2, open‐label trial to evaluate valganciclovir as a treatment to prevent development of SNHL in infants with asymptomatic cCMV infection (CASG NCT03301415) was initiated, but was terminated due to safety concerns identified during the course of the trial (https://www.clinicaltrials.gov/study/NCT03301415?tab=results). In light of this information, and absent any evidence of benefit, we do not recommend treatment of asymptomatic infants identified by universal cCMV screening programmes.

We believe that long‐term effects of valganciclovir treatment in young children remain a potential concern, such as the theoretical risks of malignancy and azoospermia [105, 106, 107, 108, 109]. We believe it is important to paint a realistic picture with families regarding the risks and benefits of valganciclovir, and to not overstate the impact of antiviral therapy. We suggest that it is important in this context to be transparent about the real and theoretical risks of valganciclovir too, including the risks of cytopenias, the risks attendant with regular laboratory monitoring, the high cost of the drug, and the possibility of emergence of antiviral resistance. We note that neutropenia can be ameliorated by concomitant administration of granulocyte colony‐stimulating factor [110, 111].

Finally, irrespective of the treatment scenario being utilised, we do not recommend monitoring of viral load as a marker of response to therapy or as a prognostic biomarker. The importance of CMV viral load at birth as a biomarker germane to treatment decision‐making or as a prognostic marker is controversial (reviewed in [112]) but there is no evidence that the patterns, rates or magnitude of viral load change while on antiviral therapy has any utility in the management of cCMV. The one exception would be if antiviral resistance were suspected, in which case determination of blood viral load can substantiate resistance and facilitate molecular testing to examine for specific resistance mutations [113].

### 3.3 Follow‐Up Care

We recommend neurodevelopmental, audiologic, and, in some cases, ophthalmological follow‐up of all infants, including asymptomatic infants, identified by universal cCMV screening. We believe this is best individualised on a case‐by‐case basis. A general recommendation is to evaluate hearing in all children with cCMV once every 3–6 months up to 1 year of age, once every 6 months from 1 to 3 years of age, and once a year from 3 to 6 years of age [114]. A case‐control study following longitudinal assessment of infants with asymptomatic cCMV found that there were no differences after 5 years of age in cases and uninfected controls in the likelihood of progression to SNHL [115]. Whether longer‐term follow‐up into later childhood, adolescence or even adulthood is required or useful remains to be studied.

We also recommend that for most children with asymptomatic cCMV, developmental assessments can be quite reasonably performed in the office by the primary care provider, using standard tools [116]. Children with moderate‐to‐severe disease, SNHL, and other CNS sequelae (cerebral palsy, developmental delay, seizure disorders, visual disturbances including cortical blindness, and neuroimaging abnormalities) require more intense neurological follow‐up, commonly with a paediatric neurologist. Most of our experience with these symptomatic cases, as already noted, was generally gained prior to the advent of universal cCMV screening. Now, with the advent of universal screening, most cCMV cases are asymptomatic. For the asymptomatic child, we do not recommend routine follow‐up with a paediatric neurologist or developmental paediatrician, but referral is warranted for children who fail to achieve typical developmental milestones or who, over time, manifest with neurological abnormalities. Fortunately, resources for developmental follow‐up are available in Minnesota, and these are outlined in Figure 2.

## 4 Future Directions

### 4.1 Diagnostics and Therapeutics

Several emerging areas in cCMV warrant consideration, particularly as primary care clinicians deal with this issue more in the future in an evolving era of universal screening. One under‐recognized and under‐diagnosed manifestation of cCMV is that of vestibular dysfunction. Signs and symptoms include vertigo, falls, delayed motor development, poor balance, spatial awareness issues, and difficulty with activities like riding a bike [117]. Vestibular disorders are difficult to screen for, and hence are commonly overlooked [118]. Vestibular dysfunction usually tracks with the severity of SNHL [119] but it may be present even in the absence of SNHL [120]. The presence of vestibular dysfunction usually is associated with maternal first‐trimester CMV infection [121]. As more information is gained about vestibular dysfunction, this level of testing may be incorporated into diagnostic evaluations for infants identified by universal cCMV screening programs.

Another often‐overlooked manifestation of cCMV infection is the impact of the virus on dental health. Children with cCMV have an increased risk of dental caries, enamel hypoplasia, and hypocalcification [122, 123]. These effects are largely defined in children with clear cCMV symptomatic disease and have not been well‐studied in the context of asymptomatic and clinically inapparent infections; nonetheless, we do recommend early and frequent evaluation by a paediatric dentist for our patients that are identified by the universal cCMV screening programme in Minnesota.

Although valganciclovir has been valuable in improving outcomes for infants with cCMV, more information is needed about whether the completely asymptomatic infant would benefit from antiviral intervention. More information is also needed about the optimal duration of antiviral therapy, including in infants with mild disease (Section 3.2). As noted above, infants with asymptomatic cCMV infection are at risk for long‐term sequelae—most notably this consists of SNHL—but other long‐term neurodevelopmental consequences are possible, and such outcomes have not been fully studied [8, 32, 124, 125]. Another risk of universal cCMV screening is that, by identifying many infants with asymptomatic infection, we may end up over‐treating infants for whom there are no tangible benefits from antiviral therapy. There are two reasons why treating children with (mild) symptoms at birth may not benefit these children. First, CMV‐related symptoms may be a consequence of a primary infection acquired in the second half of pregnancy, infections which, in the studies of Ville and Leruez‐Ville, are not associated with sequelae [7]. It is assumed that primary infections in the first trimester (or the first 20 weeks of pregnancy) are much more likely to lead to long‐term consequences. We believe, however, that later infections during pregnancy may still result in symptoms at birth; moreover, absent maternal CMV screening programmes in the US, we virtually never know the timing of maternal infection. Secondly, many of the symptoms of cCMV can also occur in the general neonatal population in infants without cCMV (e.g., microcephaly, which occurs in 2.5% of newborns [126], petechiae, and thrombocytopaenia), which may be incidental findings. The question therefore may remain as to how many of the findings at birth are specifically attributable to cCMV. Antiviral therapy is also associated with a host of toxicities (as outlined in Section 3.2), in particular neutropenia [110]. Accordingly, we also need to accelerate testing and implementation of less toxic antivirals, such as is described in NCT03728426, a study of letermovir in cCMV (https://www.niaid.nih.gov/clinical‐trials/safety‐assessment‐oral‐letermovir‐infants‐symptomatic‐congenital‐cytomegalovirus), to try to avoid the challenges (and potential risks) of using valganciclovir in these infants.

Eventually, the results from long‐term follow‐up in these universal neonatal screening cohorts could be used to personalise the duration and intensity of future care, including antiviral therapy. Hopefully, this will also provide insights into the benefits of antiviral treatment. These data could also be used for cost‐effectiveness analyses that examine the cost‐benefit of universal cCMV screening programmes [127, 128].

Other future directions that will be important to consider—not just in the context of universal cCMV screening, but in clinical management of cCMV in general—include the unresolved question of the risk of autism spectrum disorders (ASDs) in infants with cCMV [129, 130], and the known risk of labyrinthine dysfunction [117].

Finally, clarity is required on the optimal clinical sample for universal cCMV screening. Some experts advocate for saliva‐based testing [131], but concerns remain about cost, lack of infrastructure for establishment of universal newborn saliva collection, and the risk of false‐positive results due to colostrum and breast milk exposure, leading other experts to advocate for DBS screening [42, 132]. As noted throughout this review, clarity is required on this point.

### 4.2 Biomarkers to Predict Outcome

One of the most common queries we receive in clinical practice, particularly as it relates to the asymptomatic child with cCMV identified by universal screening, is ‘will my child develop a long‐term intellectual disability’? As stressed in this review, traditional teaching has been that the major disabilities associated with cCMV are encountered in children who have symptomatic disease at birth [32], whilst for asymptomatic infants, the major neurodevelopmental sequelae are related to SNHL. Viral and host factors including host genetic polymorphisms, viral loads, and viral strain variants [7, 26, 32, 112, 133, 134, 135] may play roles in predicting the severity of clinical sequelae. These are still investigational, but such assessments may eventually include assessments of viral strain genotypes to identify variants with enhanced pathogenicity, examination of CMV's impact on modification of host cytokine and chemokine responses, and studies to evaluate for virally‐mediated perturbation of the host microbiome [112]. Ouellette et al. recently examined the blood transcriptional profile of 80 infants with cCMV (49 symptomatic, 31 asymptomatic), who were being followed longitudinally for the first 3 years of life for hearing and neurodevelopmental outcomes. Transcriptome analysis of infant gene expression successfully identified a 16‐gene classifier ‘signature’ with an accuracy of > 90% that correlated with SNHL [136]. It is likely that future cCMV screening programs will be coupled with ‘second‐tier’ testing of biomarkers that, in turn, can inform clinicians and families of the likely prognostic implications of a positive cCMV screen for any given newborn and, possibly, will help inform therapy and management considerations [137].

## 5 Conclusions

As a group of paediatric infectious diseases specialists focused on the management of cCMV in this rapidly evolving period of implementation of universal screening, there seems to be a lot we don't know yet. Humility is required. The over‐arching question is what to do with the large number of healthy‐appearing newborns who have cCMV infection, but no phenotype. We are unified in our support of universal cCMV screening to connect infants to audiological follow‐up early in life. However, there are so many questions about management of infants with CICMC we just don't have great answers to yet. As we summarise in this review, all of the questions around neuroimaging, how to manage isolated laboratory findings, and how to counsel parents about prognosis are very challenging. Drug toxicity is a concern, and guidelines for management of neutropenia are challenging. Data from NCT03728426, the study of letermovir in cCMV, are needed. Should we follow viral loads in these infants, and does it have meaning? Monitoring viral load on therapy may provide a clue for the emergence of antiviral resistance which, though rare, has been described [113, 138], but does it have prognostic or predictive importance? And what about other biomarkers that may predict outcome? There also remains the fundamental question of whether cCMV screening ‘belongs’ on the newborn screening panel. Only three US states (Minnesota, New York, and Connecticut), and two Canadian provinces (Ontario and Saskatchewan) have taken this path, but the province of Quebec has gone on record as *not* supporting universal cCMV screening [https://www.inesss.qc.ca/en/publications/publications/publication/pertinence‐de‐lajout‐du‐depistage‐universel‐de‐linfection‐congenitale‐au‐cytomegalovirus‐cmv‐au‐programme‐quebecois‐de‐depistage‐neonatal.html]. We are also concerned about the shame, guilt and anxiety that can be generated for parents with cCMV screen‐positive infants [65, 139], and we need better strategies for educating pregnant people and families. Until a successful vaccine for cCMV prevention is licenced [140, 141], universal screening must be coupled to programmes that focus on enhanced knowledge and awareness of the important unmet need that this virus presents to the health and well‐being of children.

## Author Contributions

E.R.H., M.R.S. and W.C.H. all contributed to conception and design, draughting the article, and to revising it critically for important intellectual content, and gave final approval of the version to be published.

## Funding

Grant support from the National Institutes of Health (Eunice Kennedy Shriver National Institute of Child Health and Human Development), R01 HD118989, and R01 HD099866, is acknowledged.

## Conflicts of Interest

M.R.S. is site P.I. (University of Minnesota) for the Moderna mRNA‐1647 CMV vaccine trial. W.C.H. discloses ownership of stock in Pfizer, Bristol Myers Squibb, and Zimmer Biomet.

## Data Availability

Data sharing not applicable to this article as no datasets were generated or analysed during the current study.
